# Prioritisation of specialist health care services: going beyond the evidence

**DOI:** 10.1186/1472-6963-14-S2-O40

**Published:** 2014-07-07

**Authors:** Philip Webb, Khesh Sidhu, Geoffrey Carroll, Pippa Anderson

**Affiliations:** 1 Welsh Health Specialised Commissioning Committee, Caerphilly, UK; 2 Swansea University, Swansea, UK

## Background

Common approaches taken for policy formulation in the face of resource constraints are to adopt utilitarian frameworks that seek maximisation of societal health benefits. However this does not always seem to generate socially and politically palatable solutions.

## Objectives

In 2012-13 the Welsh Health Specialised Services Committee (WHSSC) developed a prioritization process for specialised health services to directly incorporates the Rule of Rescue and other psychological, emotional and social responses.

## Materials and methods

A Prioritisation Panel representing a wide range of stakeholders was convened. A master list of services was achieved through matching against criteria (including high cost individual care, growth or implementation that exceeded an incremental cost of £50,000, uncertainty about evidence or ability to benefit) for evidence and prioritisation. Condition-Treatment pairs were created for the services falling under the remit of WHSSC, evidence reviews undertaken and evidence of effectiveness and cost effectiveness were collated to inform the decision making process. Discreet choice methods were used to rank order and apply a cut off point for commissioning or not. Score cards were developed to score for scientific rigour, inclusiveness, transparency, independence, challenge, review, support for implementation and timeliness. These features relate to the procedural justice requirement for ‘accountability for reasonableness’ described in the published literature.

## Results

The common finding for the condition treatment pairs was lack of evidence to guide confident decision making. Through the process, the Panel was required to make judgements: scientific value judgements about interpreting the quality and significance of the evidence available and social value judgements. These latter were guided by four principles: respect for autonomy, non-maleficence, beneficence and distributive justice. A prioritisation and commissioning list was created and specific services identified for commissioning and decommissioning.

## Conclusions

The desire for rapid evidence assessment and policy development conflicted with the need for policy to be based on robust evidence and subject to appropriate consultation. One outcome of this programme was identification of the need to make appropriate arrangements for policy to be developed at the time it is needed without compromising its quality. Given the specialist nature of services being reviewed issues were encountered in enabling patient understanding of evidence. The policy implementation phase was also challenging. Reflecting on the experience of the process and outcomes has led to revisions for the 2014 Prioritisation Panel activities.

